# Case Report: Complete Response of Recurrent and Metastatic Cystadenocarcinoma of the Parotid Gland With a Single Course of Combined Nivolumab and Ipilimumab Therapy

**DOI:** 10.3389/fonc.2021.618201

**Published:** 2021-02-26

**Authors:** Yoshiyuki Nakamura, Masahiro Nakayama, Bungo Nishimura, Naoko Okiyama, Ryota Tanaka, Yosuke Ishitsuka, Shin Matsumoto, Yasuhiro Fujisawa

**Affiliations:** ^1^Department of Dermatology, Faculty of Medicine, University of Tsukuba, Tsukuba, Japan; ^2^Department of Otolaryngology, Head and Neck Surgery, Faculty of Medicine, University of Tsukuba, Tsukuba, Japan

**Keywords:** cystadenocarcinoma, parotid gland, nivolumab, ipilimumab, complete response

## Abstract

Although cystadenocarcinoma is classified as a low-grade histological subtype of salivary gland carcinoma (SGC), recurrence and metastases sometimes develop. However, standard treatments for advanced cases have not yet been established. Here, we present a case of unresectable local recurrence and cervical lymph node metastases of cystadenocarcinoma of the parotid gland with multiple lung nodules, all of which showed complete response with only a single course of combined nivolumab and ipilimumab therapy. The patient's medical history of metastatic melanoma roused our suspicions that the multiple lung nodules were cystadenocarcinoma metastases or malignant melanoma. Combination therapy was used based on our suspected diagnosis of lung metastases of melanoma although histological examination of the lung nodules could not be performed. While various chemotherapies are used for advanced SGCs including cystadenocarcinoma, overall, the results are unsatisfactory. In contrast, there have not yet been any reports of advanced cystadenocarcinoma of the salivary gland treated with immune checkpoint inhibitors (ICIs). Given that, in our case, a single course of combined ICI therapy induced a complete response in the unresectable and lymph node metastases from the cystadenocarcinoma and the multiple lung nodules, ICIs, including combined therapy, could be a promising treatment for advanced cystadenocarcinoma.

## Introduction

Salivary gland carcinomas (SGCs) are relatively rare, and the most common histopathological SGC types are mucoepidermoid carcinoma and adenoid cystic carcinoma (1). In contrast, cystadenocarcinoma is extremely rare, with the estimated incidence being only 2% of all SGCs (2). Although cystadenocarcinoma is classified as a low-grade histological subtype of SGC, recurrence and metastases sometimes develop (2, 3). However, standard treatments for advanced cases have not been established (3). Here, we present a case of unresectable, metastatic cystadenocarcinoma of the parotid gland with multiple lung nodules suspected to be metastatic lesions of cystadenocarcinoma or malignant melanoma, all of which showed complete response (CR) with a single course of combined nivolumab and ipilimumab therapy.

## Case Description

**Figure 1:**
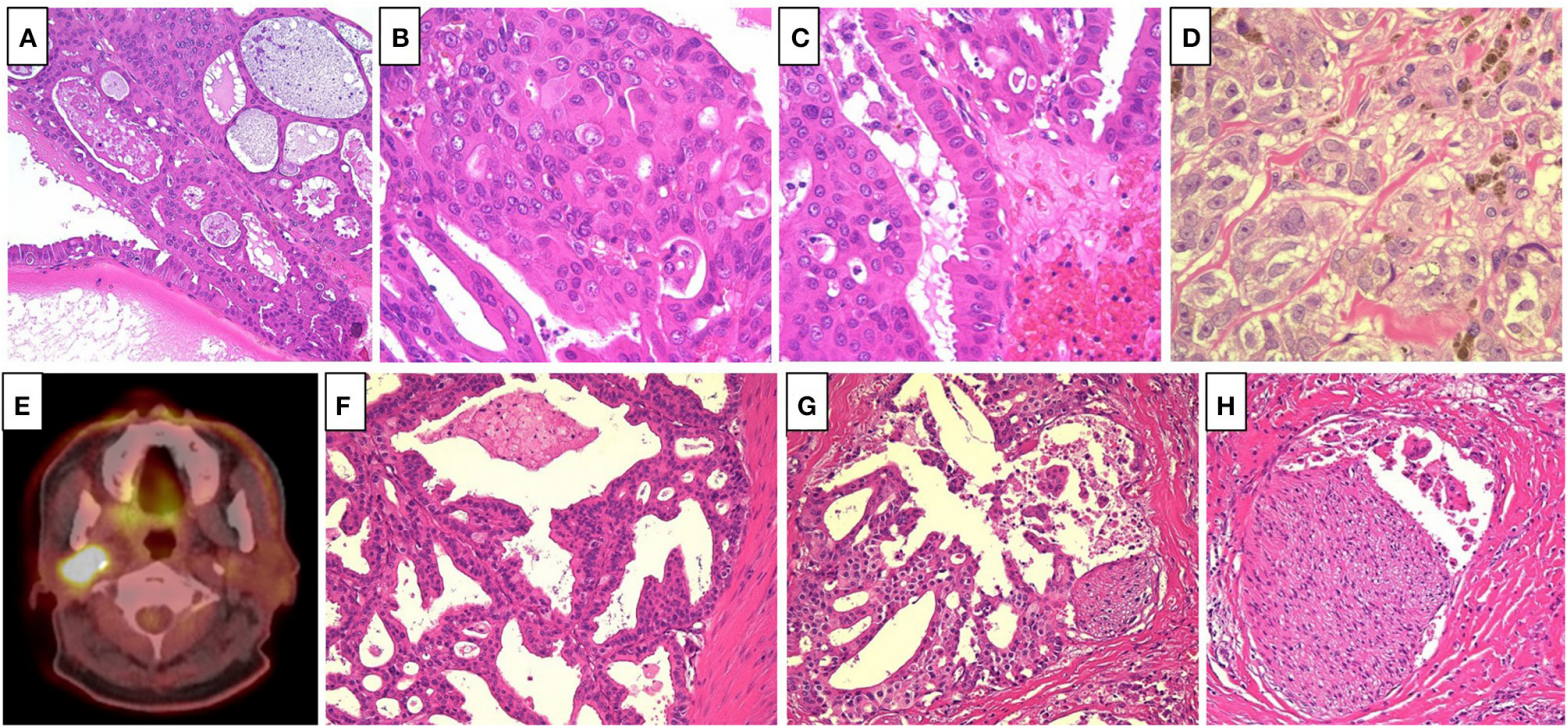
**(A)** Multiple cysts of various size lined by cuboidal, eosinophilic cells, which contained eosinophilic materials (Hematoxylin and eosin [HE], ×40). **(B)** The tumor cells showed mild to moderate atypia (HE, ×400). **(C)** Some of the tumor cells showed decapitation secretion (HE, ×400). **(D)** The cutaneous nodules revealed proliferation of large atypical epithelioid cell with melanin pigmentation, suggesting metastatic melanoma (HE, ×400). **(E)** Positron emission tomography-computed tomography revealed a mass in the deep lobe of the right parotid gland with high fluorodeoxyglucose accumulation. **(F)** Multiple cysts with frequent papillary projection consisting of the proliferation of cuboidal cells with pleomorphism (HE, ×200). **(G,H)**. Tumor nests invaded into the fibrotic stroma, and multiple perineural invasions were also observed (HE, ×200).

**Figure 2:**
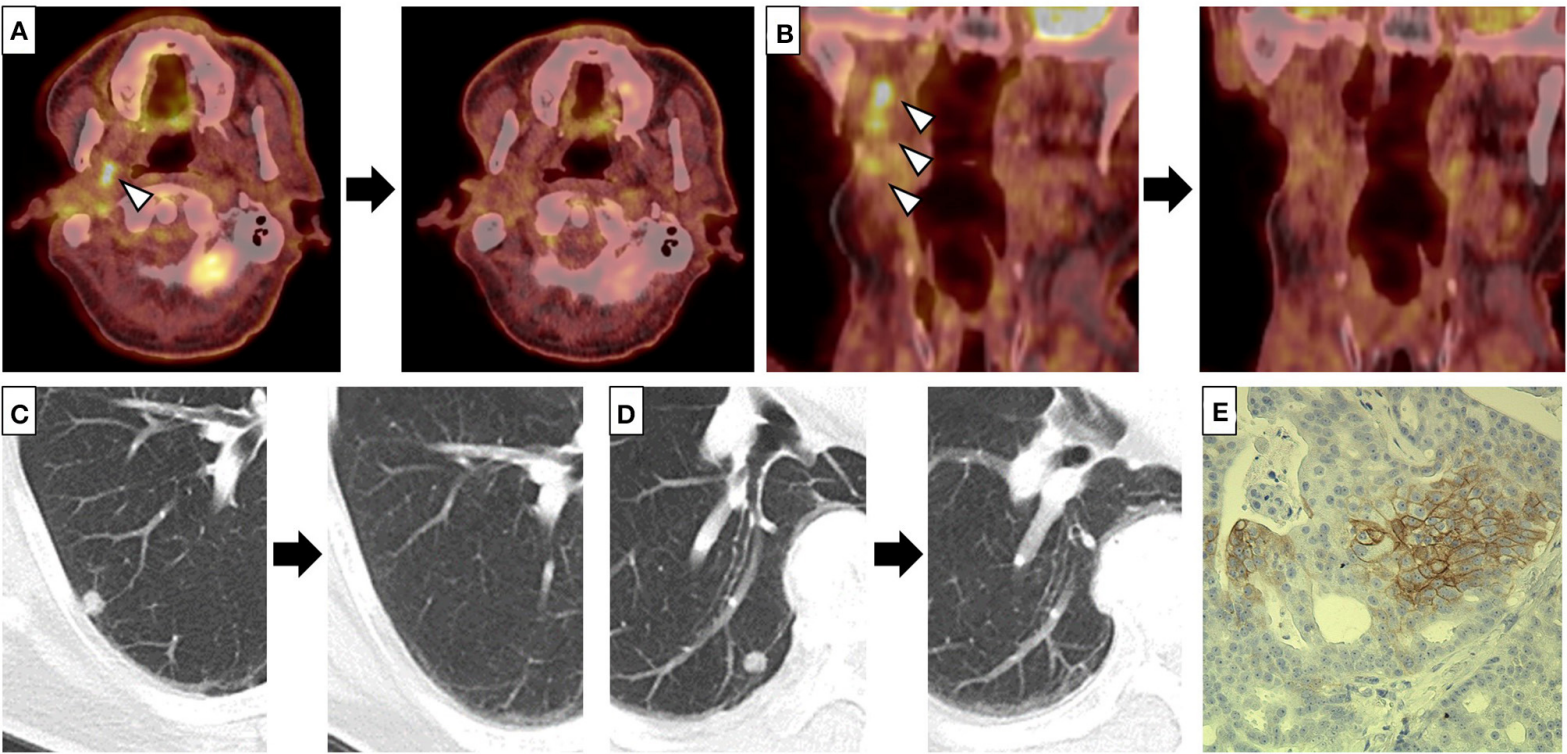
**(A–D)** Fluorodeoxyglucose (FDG) accumulation of the mass below the surgical wound of the parotid gland **(A)** and right upper deep cervical lymph nodes (LNs) **(B)**, and multiple lung nodules **(C,D)** disappeared after a single course of combined nivolumab and ipilimumab therapy. Left and right panels indicate positron emission tomography-computed tomography (PET-CT) **(A,B)** or CT **(C,D)** before and 4 months after a single course of combined therapy, respectively. Arrow heads indicate the mass below the surgical wound of the parotid gland **(A)** and the cervical LNs **(B)** with high FDG accumulation, respectively. **(E)** Some of the tumor cells of the recurrent cystadenocarcinoma were positive for PD-L1 (clone: sp142, ×200).

A 65-year old man noticed a cutaneous nodule on his left forearm, which had gradually grown. He had a medical history of diabetes mellitus and hypertension. The cutaneous nodule was resected, and was diagnosed to be malignant melanoma. He was referred to our hospital, and underwent wide resection with left axillar lymph node (LN) dissection. The surgical margin of the primary tumor was negative, and one out of the 11 dissected LNs showed metastases. 1 year after the surgery, computed tomography (CT) revealed a nodule in the right parotid gland. The nodule was resected, and histological analyses revealed multiple cysts of various sizes lined by cuboidal, eosinophilic cells with mild to moderate atypia (Figures 1A–C). The cyst lumen contained eosinophilic materials, and some of the cuboidal cells showed decapitation secretion (Figures 1A–C). There were no myoepithelial cells around the luminal cells. In some parts, the tumor cells showed microinvasion. From these findings, a diagnosis of cystadenocarcinoma was made. 4 months after the resection, a nodule with cystadenocarcinoma recurred in the right parotid gland, and right superficial parotidectomy was performed. The surgical margin was negative, and the patient received postoperative irradiation with 60Gy. At 4 and 6 years after the parotidectomy, resection was performed on two cutaneous nodules with metastatic melanoma that appeared on the left forearm (Figure 1D). PD-L1 expression level was <1%, and BRAF mutations were not detected. 9 years after the parotidectomy, positron emission tomography (PET)-CT revealed a 40 × 40 mm mass in the deep lobe of the right parotid gland with high fluorodeoxyglucose (FDG) accumulation (Figure 1E). In addition, multiple small nodules appeared in the lung. The mass in the parotid gland was resected, and histological analyses revealed multiple cysts with frequent papillary projection consisting of a proliferation of cuboidal cells with pleomorphism, indicating recurrent cystadenocarcinoma (Figure 1F). Tumor nests had invaded into the fibrotic stroma and multiple perineural invasions were also observed (Figures 1G,H). Because the mass had extended widely, it could not be completely excised and large parts of the surgical margin remained positive. 8 months after the last surgery, PET-CT revealed an irregularly shaped mass with high FDG accumulation below the surgical wound of the parotid gland (Figure 2A). In addition, right upper deep cervical LNs also showed high FDG accumulation (Figure 2B), indicating local recurrence and cervical LN metastases of cystadenocarcinoma. The size and number of lung nodules had also increased (Figures 2C,D). Because the lung nodules and the mass of recurrent cystadenocarcinoma appeared simultaneously, the nodules may be lung metastases of the cystadenocarcinoma. However, the lung nodules were also suspected to be metastatic melanoma because of the patient's medical history of lymph node and multiple skin metastases of melanoma. The patient would not agree to proposed partial lung resection for histological examination of the nodules. Therefore, based on our suspected diagnosis of lung metastases of melanoma, we proposed treatment with either anti-PD-1 antibody monotherapy or nivolumab and ipilimumab combination therapy. He selected the combination therapy, and was treated with combined nivolumab (80 mg/body) and ipilimumab (3 mg/kg) therapy. 2 weeks after the first administration of these antibodies, he developed Grade 4 liver injury, which required cessation of the treatment and a high-dose oral steroid with mycophenolate mofetil followed by steroid pulse therapy for improvement of the liver injury. However, PET-CT 4 months after the single course of the combination therapy showed that the FDG accumulation of both the mass below the surgical wound of the parotid gland and the cervical LNs had disappeared (Figures 2A,B). In addition, all of the lung nodules had disappeared (Figures 2C,D), suggesting that the combination therapy induced CR. At 10 months' follow-up after the immune checkpoint inhibitor (ICI) treatment, there was no recurrence.

## Discussion

In our case, the mass below the surgical wound and the cervical LNs with high FDG accumulation were regarded as local recurrence and metastases of the cystadenocarcinoma, respectively. However, it was unclear whether the multiple lung nodules were metastases of cystadenocarcinoma or melanoma. Management of advanced melanoma is rapidly evolving, and recent clinical trials revealed that ICIs, such as anti-PD-1 antibody (nivolumab, pembrolizumab) and anti-CTLA-4 antibody (ipilimumab), significantly prolonged the survival of melanoma patients (4–6). Despite having higher rates of adverse events (AEs), nivolumab and ipilimumab combination therapy is known to show higher efficacy compared with either agent alone (7). Based on the suspected diagnosis of metastatic melanoma of the lung and according to the patient's intent, we treated the lesions with this combination therapy. As a result, despite the occurrence of severe liver AE, only a single course of the therapy induced CR.

Cystadenocarcinoma is characterized by prominent cystic structures lined with cuboidal, columnar or mucus-secreting cells, but lacking features of other specific SGC types (8). Although most cases of cystadenocarcinoma show an indolent behavior, recurrence and LN metastases may also develop (9, 10). In addition, cases showing distant metastases such as lung and bone metastases have been reported (3). While various chemotherapies are used for advanced SGCs and their efficacy may be dependent on histological type, overall, the results are unsatisfactory and there have been no reports of significant tumor responses to systemic chemotherapies in advanced cystadenocarcinoma (1, 11–13).

Programmed death ligand 1 (PD-L1) expression in SGC tumor cells is frequently observed, indicating that it may play an important role in the immune tolerance and progression of SGC (14). On examining PD-L1 expression, it was detected in more than 5% of the tumor cells of the recurrent cystadenocarcinoma although not in the metastatic melanoma (Figure 2E). In contrast, Ross et al. analyzed tumor mutation burden (TMB), which is known to be associated with favorable tumor response to ICIs, in 623 cases of SGC, and reported that the TMB of SGC was significantly lower than that of other tumor types for which ICIs were approved such as melanoma and breast cancer, although cases of cystadenocarcinoma were not included in this study (15). Consistently, recent studies demonstrated limited efficacy of anti-PD-1 monotherapy for advanced SGCs (16–18). However, there were no cases of cystadenocarcinoma within these studies, and there have been no reports of advanced cystadenocarcinoma of the salivary gland treated with ICIs. In our patient, the recurrent tumor of the parotid gland and cervical LN metastases of cystadenocarcinoma as well as the multiple lung nodules achieved CR with only a single course of combined nivolumab and ipilimumab therapy. Thus, our case suggests that ICIs, including combined therapy, could be a promising treatment for advanced cystadenocarcinoma.

## Data Availability Statement

The raw data supporting the conclusions of this article will be made available by the authors, without undue reservation.

## Ethics Statement

This patient has provided written, informed consent for publication. A copy of the signed consent form is available on request.

## Author Contributions

YN, MN, and BN designed the study. NO, RT, YI, SM, and YF interpreted the results. YN and MN wrote the manuscript. All authors reviewed the manuscript.

## Conflict of Interest

The authors declare that the research was conducted in the absence of any commercial or financial relationships that could be construed as a potential conflict of interest.
